# Differential Expression Pattern of Retroviral Envelope Gene in the Equine Placenta

**DOI:** 10.3389/fvets.2021.693416

**Published:** 2021-07-09

**Authors:** Valentina Stefanetti, Luisa Pascucci, Sandra Wilsher, Katia Cappelli, Stefano Capomaccio, Lara Reale, Fabrizio Passamonti, Mauro Coletti, Martina Crociati, Maurizio Monaci, Maria Luisa Marenzoni

**Affiliations:** ^1^Department of Veterinary Medicine, University of Perugia, Perugia, Italy; ^2^The Paul Mellon Laboratory of Equine Reproduction, ‘Brunswick’, Newmarket, United Kingdom; ^3^Department of Agricultural, Food and Environmental Sciences, University of Perugia, Perugia, Italy; ^4^Centre for Perinatal and Reproductive Medicine, University of Perugia, Perugia, Italy

**Keywords:** horse, placenta, equine endogenous retrovirus, RT-qPCR, *in situ* hybridization

## Abstract

Endogenous retroviruses (ERVs) are proviral phases of exogenous retroviruses, which have coevolved with vertebrate genomes for millions of years. The conservation of ERV genes throughout evolution suggests their beneficial effects on their hosts' survival. An example of such positive selection is demonstrated by the syncytin gene, which encodes a protein with affinity for various mammalian placentas that is involved in the formation of syncytiotrophoblasts. Although the horse has an epitheliochorial placenta, in which the fetal trophoblasts are simply apposed to the intact uterine epithelium, we have previously demonstrated that the equine ERV (EqERV) *env* RNA is unexpectedly expressed in placental tissue. In the present study, we investigated the mRNA expression pattern of the EqERV *env* gene in different parts of the equine placenta, to gain more insight into its putative role in the fetal–maternal relationship. To this end, we used reverse transcription–quantitative PCR (RT–qPCR) and *in situ* hybridization assays to analyze different target areas of the equine placenta. The retroviral *env* gene is expressed in the equine placenta, even though there is no syncytium or erosion of the uterine endometrium. The gene is also expressed in all the sampled areas, although with some quantitative differences. We suggest that these differences are attributable to variations in the density, height, and degree of morphological complexity of the chorionic villi forming the microcotyledons. The involvement of the EqERV *env* gene in different functional pathways affecting the fetus–mother relationship can be hypothesized.

## Introduction

Retroviruses are characterized by their ability to integrate their DNA into the genomes of their host cells, forming so-called “proviruses” (1). When this integration occurs in a germ-line cell, the provirus is transmitted vertically in a typical Mendelian fashion and becomes fixed in the host genome as an endogenous retrovirus (ERV) (2). This type of virus, which is present in virtually all vertebrate genomes, is considered a “genomic fossil record,” from which the long-term interactions between retroviruses and their hosts can be inferred (3). Most ERVs have not been subject to selective pressure, and have been progressively disrupted by the accumulation of deletions or mutations, which destroy their coding capacity (4). For decades, ERVs have been considered “junk DNA” that has no effect on the evolution of their host (5). However, ERVs would have become progressively extinct if their expression had exerted deleterious effects on the host organism. On the contrary, the abundance of these elements in host genomes indicates that they play a role in genomic plasticity. Furthermore, the presence of transcriptionally active ERVs with intact open reading frames (ORFs), conserved for millions of years after their integration, suggests that some ERVs have been co-opted by the host for specific biological roles (6). The syncytin genes, which encode the ERV envelope (Env) protein, the fusogenic activity of which is required by exogenous retroviruses to gain entry into host cells, appear to have a particular affinity for the placentas of various mammals, including Primates (7), Muridae (8), Lagomorpha (9), Carnivora (10), Ruminantia (11), Afrotherians (12), and even the distantly related Marsupialia (13). These syncytins have been well-characterized and are associated with the invasive types of placentation, where they are involved in the formation of syncytiotrophoblasts. The various syncytin genes have been integrated independently into their mammalian host genomes and subsequently adapted for a role in placentation during mammalian evolution. Therefore, it has been proposed that this stochastic acquisition of exogenous genes participated in creating the structural and functional diversity of the mammalian placentas (14).

Although the equine reference genome became available in 2007, information about equine ERVs remains limited. The few *in silico* studies that have analyzed ERVs in the horse genome have detected a completely intact provirus (5′-long terminal repeat [LTR]-gag-pro-pol-env-3′-LTR), formally designated “EqERV-beta1” (15), and a further 978 sequences have been annotated as potential ERVs (16). Another study reported a total of 1,947 putative ERVs when three analytical methods were compared (17). Recently, Zhu and colleagues used a range of bioinformatic approaches to characterize the ERV diversity in the perissodactyl genome, establishing that at least nine different genome-invasion events have occurred in the perissodactyl lineage and that all nine ERVs are still present in equids (18). Only a few studies have examined the expression of EqERVs in tissues other than reproductive tissues (16, 19). Although the horse has an epitheliochorial placenta, in which the fetal and maternal tissues are simply apposed to one another, with no syncytiotrophoblast formation, we showed in a previous study that the EqERV *env* RNA is more strongly expressed in the horse placenta than in other somatic tissues, as might be expected for a candidate syncytin-like gene (20). We proposed that the intact EqERV *env* gene is a candidate syncytin-like gene, as identified by Zhu et al. (18), and that it belongs to the zeta lineage.

The equine placenta is classified as diffuse, non-invasive, and epitheliochorial, although part of the early preimplantation placenta, the chorionic girdle, does invade the endometrium to form the endometrial cups that are unique to equines. The major portion of the placental membranes consists of the allantochorion, which derives from the fusion of the allantois with the chorion. The chorionic surface of the allantochorion is covered by microcotyledons, each of which is composed of tufts of branched well-vascularized villi that interdigitate with the maternal uterine crypts, which are lined with simple epithelium (21). Although the allantochorion looks grossly homogeneous across its chorionic surface, with a red velvety appearance, closer inspection reveals differences between the different areas, depending upon their positions in the uterus and the position of the fetus. Because the allantochorion occupies the whole of the uterus, it mimics its shape, with two horns and a body portion. The placental horn that contains the fetus is called the “gravid horn” and is usually thinner, with more widely spaced microcotyledons, than the non-gravid horn, which has longer and denser microcotyledons (22, 23). These differences are probably due to the degree of distension of the different areas by the fetal fluids and the fetus itself. A few small areas on the chorionic surface of the allantochorion lack villi. These avillous regions overlie areas that are opposite regions not in direct contact with the glandular endometrium: the internal os of the cervix (known as the “cervical star” on the allantochorion), the uterotubal junctions at the tip of each uterine horn, and a ring around the cord attachment site, where the chorion has overlain the endometrial cups before their regression. Scarring of the endometrium or folding of the chorion (for example over major placental vessels) also generates avillous areas on the chorionic surface (22, 24). The other major placental membrane is the allantoamnion, a fusion of the allantois and amnion, which surrounds the fetus to form the amniotic cavity. This shiny, translucent membrane is rich in vessels that originate from the cord. The umbilical cord itself is usually attached dorsally to the allantochorion and has an intra-allantoic and intra-amniotic portion (22). In our previous study, an initially unrecognized bias in the sampling process was hypothesized because the placental samples were collected randomly from the placental membranes and pooled in a non-systematic way. This was probably responsible for the widely heterogeneous results, as demonstrated with a variance analysis (20).

The present study was undertaken to determine the mRNA expression pattern of the EqERV *env* gene more precisely in different portions of the equine placenta, to gain insight into its role in the fetal–maternal relationship. To this end, reverse transcription–quantitative PCR (RT–qPCR) and *in situ* hybridization (ISH) assays were used to analyze targeted samples from different areas of 12 full-term equine placentas.

## Materials and Methods

### Sample Collection

The placentas of 12 mares, aged 4–10 years (mean, 5.8 years), were collected within 2–4 h of expulsion following a eutocic delivery at the end of a physiologically normal gestation period (330–350 days). All samples were collected during routine post-delivery clinical examinations by licensed equine veterinarians on private stud farms, with the written informed consent of the owners and in accordance with Ethical National and European regulations.

To investigate EqERV *env* expression in the equine placenta, seven defined areas of the placenta were sampled: the villous allantochorion next to the cervical star (S1); the villous allantochorion next to the site of attachment of the umbilical cord (S2); the villous allantochorion in the gravid horn (S3) and the non-gravid horn (S4); the avillous cervical star (S5); the avillous area of the allantochorion equatorially around the umbilical cord attachment site (S6); and the amnion (S7). The spatial relationships of these parts are shown in the Supplementary Figure 1. Fetal membranes that showed macroscopic lesions or histological abnormalities were excluded from the study.

Three samples were collected from each placental area: two were immediately frozen in liquid nitrogen and stored at −80°C until RNA extraction, and the third was fixed in 10% neutral-buffered formalin for 24 h at 4°C and embedded in paraffin for ISH analysis.

### RT-qPCR

Total RNA was extracted from 100 mg of ground tissue from each sample using the Trizol Plus RNA Purification Kit (Ambion, Life Technologies, Monza, Italy), according to the manufacturer's instructions. The total RNA (1 μg) was treated with DNase I (Amplification Grade, Invitrogen), quantified with a NanoDrop2000® spectrophotometer, and its integrity examined with electrophoresis in a denaturing 1% agarose gel.

The DNase-treated RNA (400 ng) was reverse transcribed into cDNA with a mixed priming strategy (oligo[dT] and random hexamers) using the PrimeScript RT Reagent Kit (Takara), according to the manufacturer's instructions. The qPCR reaction was performed with 5 μl of the diluted (1:10) cDNA in a final volume of 20 μl, using 10 μl of SsoFast™ EvaGreen® Supermix (Bio-Rad, Hercules, CA, USA) and an already published primer pair (20). Amplification was performed in a CFX96 Touch Real-Time PCR system (Bio-Rad). Each reaction was run in triplicate and no-template controls were included in each run. The successful removal of the genomic DNA was confirmed by directly amplifying the RNA extracts without reverse transcription. The specificity of the PCR product was determined with a melting curve analysis, by checking the amplicon size in agarose gel, and with DNA sequencing.

Six different reference genes were also tested to select the most stable genes for the normalization of the data: ribosomal protein L32 (*RPL32*), β-actin (*ACTB*), ribosomal RNA 18S (*18S*), hypoxanthine phosphoribosyltransferase (*HPRT*), beta-2 microglobulin (*B2M*), and glyceraldehyde-3-phosphate (*GAPDH*). The corresponding primer pairs are listed in the Supplementary Table 1. The six candidate reference genes were analyzed with the Reference Gene Selection Tool (Bio-Rad). This software uses the GeNorm algorithm, which calculates a statistical measure of stability for each pair of reference genes (also known as the “M value”) and provides a ranking of the tested genes based on their stability measurements.

The expression ratio of the genes of interest was normalized to the relative abundance of the two reference genes using the ΔΔ Cq method (25), which uses the geometric mean of quantification cycles (Cq) values. The data were analyzed with the Bio-Rad CFX Manager software (ver. 3.2.2) and GenEx (ver.6).

Differences between different areas of the placentae were analyzed by ANOVA test, and changes in the relative gene expression between the groups were calculated using a *t* test. *P* ≤ 0.05 were considered statistically significant. Expression values are presented as means of fold change (±sem) calculated using CFX maestro software (ver. 4.1- BioRad, Hercules, CA, USA).

### Probe Synthesis and *in situ* Hybridization

Two probes were synthesized for ISH. The antisense RNA probe, which is a sequence of single-stranded RNA complementary to the coding sequence of target mRNA, and the sense RNA probe, with the same sequence as the mRNA, generated to be used as a negative control in order to assess the degree of non-specific hybridization. PCR-amplified EqERV *env* fragment of 540 bp (Primers: 5′-CTCCCAGGCCACAATCAAAC- 3′ and 5′-CCAGTGGAAGACAGAGAGGG-3′) was cloned into Topo TA cloning (ThermoFisher Scientific) for *in vitro* synthesis of the probes. The orientation of the insert was evaluated by PCR assay using T3 and T7 primers as per the manufacturer's recommendation and confirmed by Sanger sequencing. According to the orientation, the antisense and sense riboprobes were generated with T3 and T7 RNA polymerase and DIG RNA labeling mix (Roche Applied Science), used to add digoxigenin-UTP nucleotides into RNA, which allowed detection with an anti-DIG antibody.

After the reaction, the RNA transcripts were analyzed by agarose gel electrophoresis and ethidium bromide staining. The efficiency of the labeling was evaluated by a dot blot assay, by applying a series of dilutions of DIG-labeled RNA probe to a positively charged nylon membrane and performing an immunological detection reaction on the membrane with alkaline phosphatase-conjugate anti-digoxigenin antibody. Serial sections (5 μm) of four areas of each placenta were used for ISH to compare the expression of the *env* gene in the villous and avillous areas with that in the amnion. The four sites tested were the villous allantochorion next to the site of attachment of the umbilical cord (S2), the cervical star (S5), the avillous allantochorion next to the site of attachment of the umbilical cord (S6), and the amnion (S7). All sections were deparaffinized twice in xylene, and then hydrated through an ethanol series. The slides were then digested with proteinase K (1 μg/mL) for 20 min at 37°C and finally washed with cold water to stop the enzyme activity. The sections were acetylated, washed, dehydrated in an ascending series of ethanol, and air-dried. The digoxigenin (DIG)-labeled RNA probe was diluted (300 ng/μl) in hybridization buffer and the sections were hybridized at 43°C overnight. Three stringent washes were then performed at 55°C. The slides were then incubated at room temperature for 2 h with an alkaline-phosphatase-conjugated anti-DIG antibody (Vector Laboratories). The antibody was visualized with the alkaline phosphate substrates nitroblue tetrazolium (NBT) and 5-bromo-4-chloro-3-indolyl phosphate (BCIP), as instructed by the manufacturer (Roche Applied Science), and observed with light microscopy.

## Results

### Differential EqERV *env* Expression Detected With RT-qPCR

**Figure 1:**
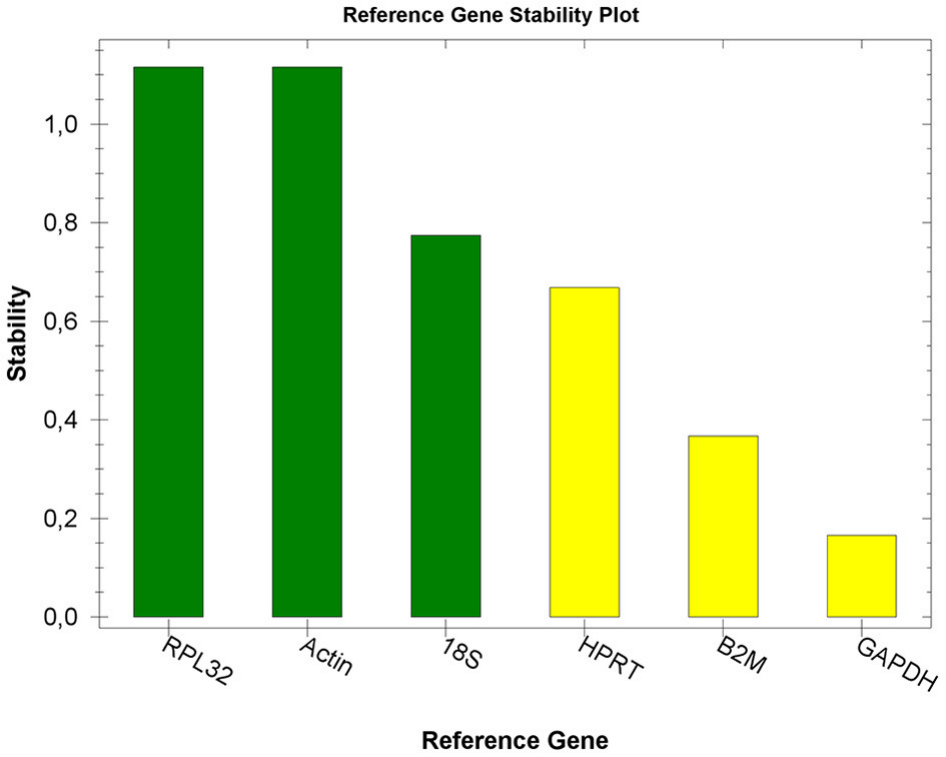
Reference gene stability plot. Expression stability values (M) of six putative reference genes tested in placental tissues. Transcripts are ranked from the most stable to the least stable (left to right).

For optimal accuracy, we performed a reference gene study specific for the equine placenta. As shown in Figure 1, *RPL32* and *ACTB* were the reference genes most stably expressed across all the samples in the experiment. Therefore, the expression of the *env* gene was normalized to the expression of these two reference genes to adjust for unbalanced samples and to correct for co-expression in the RT-qPCR.

**Figure 2:**
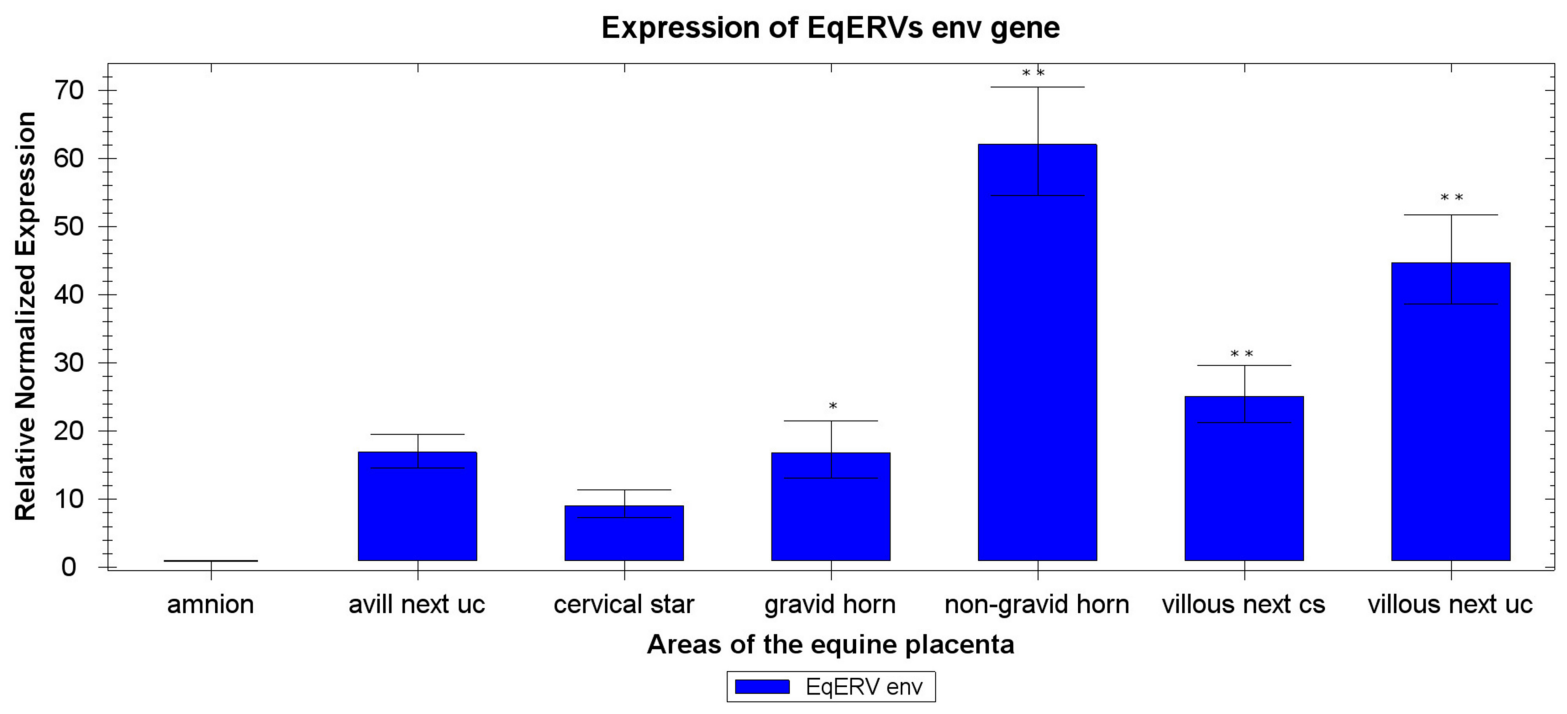
Expression of EqERVs *env* RNAs in various areas of the equine placenta detected with RT-qPCR. Each value was normalized to the levels of two reference genes (*ACTB* and *RPL32*) and is shown as the mean of triplicate experiments. The *x*-axis shows the different areas of the equine placenta, and the *y*-axis shows the relative normalized expression, with the standard error. Technical replicates were averaged and only those samples with a standard error lower than 0.2 Cq were retained. **indicates *p* < 0.001 and *indicates *p* < 0.05 with respect to amnion, as shown by ANOVA. Uc, umbilical cord attachment; cs, cervical star.

The mRNA expression levels of *env* are shown in Figure 2. This gene was expressed globally in the equine placenta, although the expression pattern varied across the different placental areas. We used the area with the lowest expression, the amnion, as the control sample: *env* mRNA was significantly higher expressed in samples from all other areas when compared with the amnion. Based on a *t* test, EqERV *env* mRNA expression differed significantly between the amnion and the villous allantochorion next to the site of attachment of the umbilical cord (*P* < 0.001), the non-gravid horn (*P* < 0.001), the gravid horn (*P* = 0.015), and the villous allantochorion next to the cervical star (*P* < 0.001). The highest expression of the *env* gene was observed in the allantochorion of the non-gravid horn, and differed significantly from that in the allantochorion of the gravid horn (*P* < 0.001), the villous allantochorion next to the cervical star (*P* < 0.001), the cervical star (*P* < 0.001), and the avillous area of the allantochorion equatorially around the umbilical cord attachment site (*P* < 0.001). The expression of *env* in the villous allantochorion next to the site of attachment of the umbilical cord also differed significantly from that in the gravid horn (*P* = 0.001), the villous allantochorion next to the cervical star (*P* = 0.03), the cervical star (*P* < 0,001), and the avillous area of allantochorion equatorially around the umbilical cord attachment site (*P* < 0.001). The expression of *env* also differed significantly between the cervical star and the villous allantochorion located next to the cervical star (*P* = 0.04).

### *In situ* Hybridization

**Figure 3:**
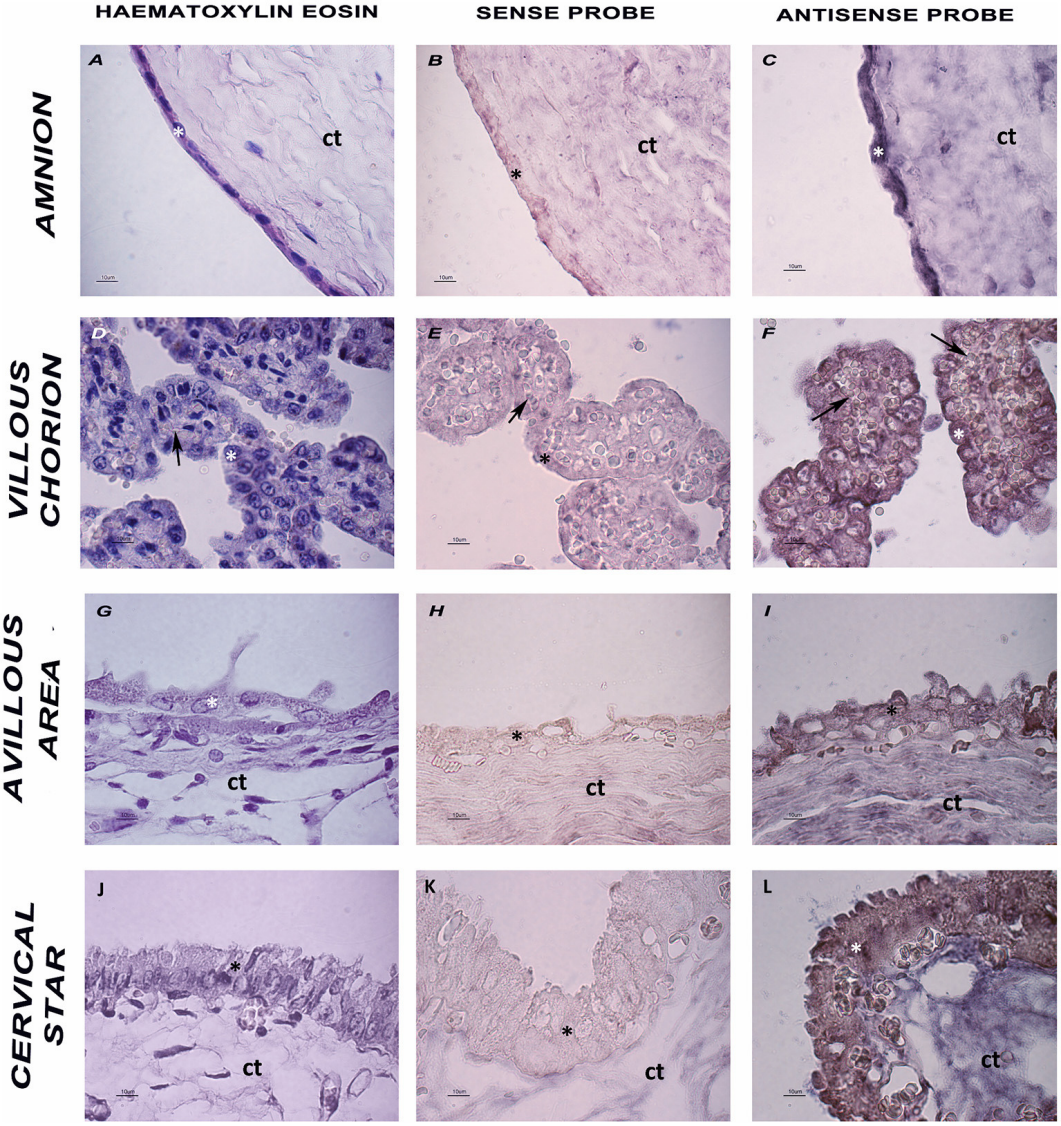
Histology and ISH of placental sections. **(A,D,G,J)** Hematoxylin and eosin staining of the four tested areas. **(B,E,H,K)** ISH negative control, sense probe. **(C,F,I,L)** ISH-Purple staining indicates the presence of EqERV *env* detected by the antisense probe. Ct: connective tissue; asterisk: cell layer lining the chorionic surface and the amnion; arrow: blood vessels. Representative images at high magnification are shown (scale bar 10 um).

An ISH analysis was performed to further characterize *env* expression in the horse placenta, and to specifically localize the *env* transcripts in the different placental areas. A specific DIG-labeled antisense riboprobe was successfully synthesized to detect the EqERV *env* transcript, and the corresponding sense riboprobe (with the same sequence as the mRNA) was also generated for use as the negative control to assess the degree of non-specific hybridization. As shown in Figure 3, labeling was only observed in the samples treated with the antisense probe, thus demonstrating the specificity of the probe. All four tested areas, representing tissues with different *env* expression, showed the presence of the *env* transcript. ISH showed a complete qualitative correspondence with the PCR-based expression analysis, confirming that *env* is expressed throughout the equine placenta. In particular, specific purple-blue labeling was detected in the cells on the outermost surface of the chorionic villi and on the amnion, with similar signal intensities in the different sampled areas. In all the sampled areas that included allantochorion, the chorionic surface of this membrane showed intense positive labeling on both the microcotyledonary villi and the absorptive areolar areas between them.

## Discussion

In eutherian mammals, the placenta is generally classified based on the number and nature of the layers at the feto–maternal interface: epitheliochorial (horses and pigs), synepitheliochorial (ruminants), endotheliochorial (carnivores), or hemochorial (mice, rabbits, and humans). In the three latter types of invasive placentas, the *env* genes of retroviral origin have been “domesticated” to play a specific role in the formation of syncytiotrophoblasts, mediated by the fusogenic property of the encoded protein (4).

However, we have previously demonstrated that this gene is unexpectedly expressed in the equine epitheliochorial placenta, in which the fetal trophoblasts are simply apposed to the intact uterine epithelium, suggesting its more complex involvement in the fetal–maternal interaction. However, the variability in the expression of *env* in different components and areas of the equine fetal membranes was not assessed in that study (20). In the present study, we undertook a more detailed investigation of EqERV *env* RNA expression in specific areas of the equine full-term placenta in order to determine whether its expression varies and to clarify the possible functions of Env in this species.

Normalization procedures were used to correct for any variation in the amount of starting material in the RT reaction, the efficiency of the RT reaction, the pipetting accuracy, and the presence of PCR-inhibitory substances in the samples (25). In our previous study (20), we used *ACTB* and *GAPDH* as reference genes, based on their use in previously published papers (26–28). In the present study, *ACTB* was confirmed as one of the two best reference genes, together with *RPL32*, whereas *GAPDH* was less stable, although it still resulted in an acceptable range. RT–qPCR indicated that EqERV *env* RNA was expressed in the placentas of all the mares studied. The expression level was the lowest in the amnion, consistent with previous studies (9, 29).

The highest expression of this gene was observed in the areas of the allantochorion that have the densest microcotyledons: the non-gravid horn and the allantochorion near the site of the cord attachment. Although the chorionic surface of the allantochorion situated in the gravid horn is also covered in microcotyledons, *env* expression was significantly lower here than in the other villous areas of the allantochorion. A functional explanation cannot be excluded, although we assume that the differences identified between the gravid and non-gravid horns may be related to the fact that the pressure exerted on the allantochorion by the fetal fluids and the fetus itself is greatest in the gravid horn, which tends to make the villi less dense and shorter than the tall, densely packed villi in the non-gravid horn (22, 23). The significantly lower expression of the EqERV *env* gene in the samples from areas devoid of microcotyledons confirms its preferential expression in microcotyledonary villi.

Baba and colleagues examined the expression of theBERV-K1 *env* mRNA of an endogenous betaretrovirus in the bovine placenta with qPCR and demonstrated that it is preferentially expressed in the placentomes (fetal cotyledons and maternal carunculae), which are the fetoplacental units in the bovine placenta in which the fetal and maternal cells fuse to form bi- and trinucleate cells (30, 31). However, the same authors also found high expression of this gene in the intercotyledonary and intercaruncular allantochorion, where cell-to-cell fusion rarely occurs (32, 33).

The *env* RNA and Env protein of endogenous Jaagsiekte sheep retrovirus (enJSRV), a betaretrovirus, were also found in the mononuclear trophoblasts of the ovine placenta, although they were most abundant in the syncytiotrophoblasts within the placentomes (34). These data support the main findings of the present study, in which the retroviral *env* gene was detected in the equine placenta, even though no syncytium or erosion of the uterine endometrium occurs. The gene was also detected in both the villous and avillous areas, although with some quantitative differences.

ISH confirmed the presence of *env* transcripts in the horse placenta. The *env* transcript was detected in all the sampled areas of the epithelium, corresponding qualitatively with the PCR-based expression analysis. However, because the ISH assay cannot quantify RNA transcript levels, we can only speculate that the differences in *env* expression in the various areas of the placenta when observed with RT–qPCR and ISH are attributable to variations in the density, height, and degree of morphological complexity of the chorionic villi that form the microcotyledons.

Based on these results, we cannot exclude the possibility that EqERV Env retains its fusogenic activity in early gestation in the one part of the equine placenta that is invasive, the chorionic girdle and the subsequently formed endometrial cups, although this is not true syncytiotrophoblast formation. The endometrial cup reaction begins around days 35–38 of gestation when a band of highly invasive chorionic girdle cells becomes detached from the fetal membranes and penetrates the maternal endometrial stroma, which they are apposed against to form the endometrial cups. These transitory endocrine structures are composed of binucleate trophoblast cells, and although they share many features with the syncytiotrophoblasts of the human placenta (35), they do not form a true syncytium in terms of the fusion of cells. At term, the endometrial cups have degenerated and the only sign of their presence on the overlying allantochorion is an avillous area around the site of attachment of the umbilical cord. As suggested earlier, the invasive nature of the chorionic girdle cells as they form the endometrial cups makes them a likely site at which EqERV *env* RNA is expressed. Because these structures are transient in early pregnancy, it was not possible to include this tissue in the present study. However, preliminary immunohistochemical studies of both the chorionic girdle and endometrial cup tissues using an antibody raised against syncytin-1, the protein encoded by the *HERV-W* gene, showed little expression in these invasive components of the equine placenta, in contrast to strong staining for syncytin-1 in the trophectoderm on day 19 and in the trophoblast of the term placenta (S. Wilsher and W.R. Allen, unpublished data).

The expression of EqERV e*nv* in all the sampled areas of the non-invasive equine placenta suggests that Env is involved in functions other than trophoblast invasion and syncytium formation. In Caviomorpha, for example, a syncytin-like gene specifically expressed in the invasive trophoblast-containing junctional zone has been identified. However, *in vitro*, the encoded protein lacks one of the canonical characteristics of syncytin, its fusogenic activity (29). Experiments on loss-of-function enJSRVs expressed in the cotyledonary portion of the placentomes in the ovine placenta also demonstrated reduced trophoblast proliferation, which prevented implantation, although the ability of Env from different enJSRVs to induce cell fusion and the formation of syncytia has not been reported (36, 37). The protein encoded by a recently identified human ERV envelope gene called *HEMO*, which has a full-length protein-coding sequence, shares some properties with syncytins, in that it is highly expressed in the placenta, but it shows no evidence of fusogenic activity (38). Overall, these examples reinforce the notion that cell-cell fusion activity is only one of the functions of *env* genes expressed in placentae. At least three other classical roles of ERV-derived proteins have been described, which are not mutually exclusive activities. These include the suppression of maternal immunity through an immunosuppressive domain at the protein level; a receptor interference mechanism that protects against exogenous viral infection; and the modulation of cell differentiation and proliferation (14).

We can only speculate about the role of EqERV Env, but our results suggest a possible involvement of this protein in the regulation of feto–maternal tolerance, a crucial requirement of pregnancy because the placenta is essentially an allogenic tissue, expressing both maternal and paternal genomes, and should theoretically be rejected by the mother. Immunosuppressive activities have been demonstrated for mouse syncytin-B (39, 40), and human syncytin-2 (41) and these functions are basically mediated by the presence of an immunosuppressive domain in the protein. Immunosuppressive activity may even be the primordial function of the syncytins, preceding the evolution of their fusogenic ability (4).

It should be emphasized that even ERVs that do not encode proteins can play important physiological roles via epigenetic regulatory systems, or by expressing long non-coding RNAs (lncRNAs). Interestingly, recent studies have suggested that ERVs have had a universal role in the evolution of the placenta, acting as non-coding regulatory elements (42). It is noteworthy that many of the ERVs identified in the study by Zhu et al. (18) overlap lncRNAs within the genome, indicating a possible role for equine ERVs in lncRNA-mediated gene regulation (43).

The high fetal *env* transcript levels described in the present study were detected in term placentas collected after a eutocic delivery at the end of a physiologically normal gestation. The possibility cannot be excluded that the time of sampling played a role in determining the level of ERV *env* expression, as demonstrated in rabbits, in which the placenta-specific expression of *env* decreases with gestational age (9). Syncytyn-Car1 transcript levels were found to be high in the term placenta of a California sea lion recovered soon after birth (10).

Several mechanisms should also be investigated to understand whether the dysregulation of genes of retroviral origin plays a role in pathological conditions. Because the morphology and density of the microcotyledons on the chorion surface of the equine allantochorion affect the expression of the *env* gene, suboptimal placentation could potentially influence its expression. For example, the development of the microcotyledons is attenuated in older subfertile mares compared with that in young fertile mares at equivalent stages of pregnancy (44). Furthermore, even in fertile mares, age and parity influence microcotyledon development (45). Therefore, it would be interesting to examine the role of this protein in mares of varying age, parity, and fertility. In conclusion, the EqERV *env* gene is expressed in the equine placenta, with higher expression in villous areas. However, the role of the encoded protein in the equine placenta remains unclear, although it may be involved in one or more physiological and/or pathological conditions. Additional experiments should help to characterize the role and function of this syncytin-like protein in the horse placenta and pregnancy.

## Data Availability Statement

The raw data supporting the conclusions of this article will be made available by the authors, without undue reservation.

## Ethics Statement

Ethical review and approval was not required for the animal study because all placental sample collections were performed during routine post-delivery clinical examinations by licensed equine veterinarians on private studfarms in accordance with Ethical National and European regulations. Written informed consent was obtained from the owners for the participation of their animals in this study.

## Author Contributions

VS and MLM: conceptualization. VS: methodology. MCr and MM: sample selection and clinical evaluations. KC, SC, and VS: validation of Rt-qPCR. LP and LR: validation of ISH. VS and FP: formal analysis. VS, LP, and MLM: writing—original draft preparation. SW and MLM: writing—review and editing. SW: supervision. LP, FP, MCo, MM, and MLM: funding acquisition. All authors contributed to the article and approved the submitted version.

## Conflict of Interest

The authors declare that the research was conducted in the absence of any commercial or financial relationships that could be construed as a potential conflict of interest.
