# Supplementary material for: Differential Expression Pattern of Retroviral Envelope Gene in the Equine Placenta
Source: Front Vet Sci. 2021 Jul 9;8:693416. doi: 10.3389/fvets.2021.693416 (PMC8298818; doi:10.3389/fvets.2021.693416)
Supplement: Supplementary file 1 [file Data_Sheet_1.pdf]

# *Supplementary Material*

## 1 Supplementary Data

Figure S1: Gross image of equine full-term placenta. The three major tissues of the equine fetal placenta are shown in this photograph: the umbilical cord, the amnion and the large chorioallantois.

Photo modified from Library of Reproduction Illustration (LORI) website, with permission of Dr Rob Lofstedt, University of Prince Edward Island.

http://loriequinesection.blogspot.com/2014/04/equine-placental-gross-anatomy-keywords.html

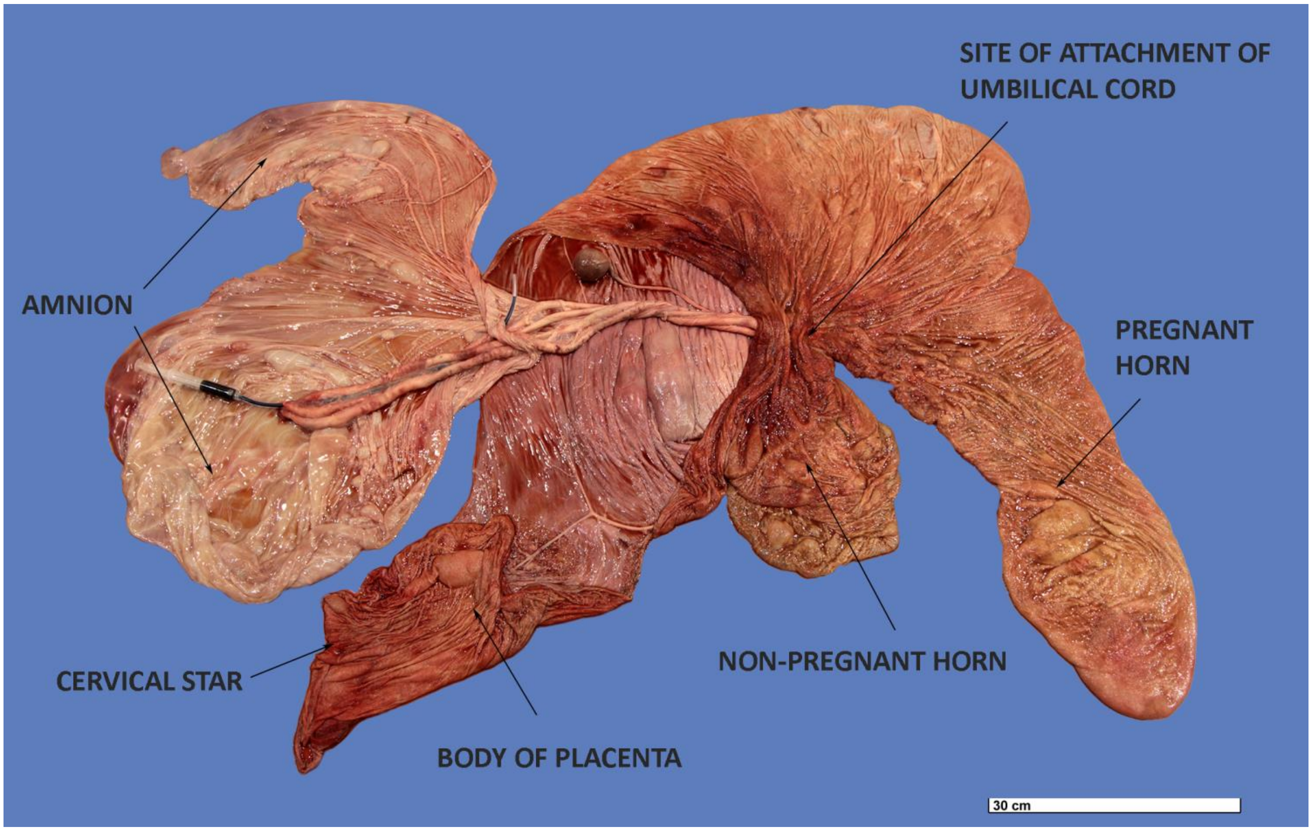

**Supplementary Table 1:** Primer sequences of the candidate reference genes.

| Gene symbol | Primer sequence (5'-3') Fw/Rev | Amplicon Lenght | Reference |
|---|---|---|---|
| *B2M* | GGCTACTCTCCCTGACTGG<br>ACACGGCAACTATACTCATCC | 271 | Kayis S.A et al., 2011 |
| *18S* | ATGCGGCGGCGTTATTCC<br>GCTATCAATCTGTCAATCCTGTCC | 204 | Kayis S.A et al., 2013 |
| *RPL32* | AGCCATCTACTCGGCGTCA<br>TCCAATGCCTCTGGGTTTC | 149 | Kayis S.A et al., 2015 |
| *GAPDH* | ATCACCATCTTCCAGGAGCGAGA<br>GTCTTCTGGGTGGCAGTGATGG | 341 | Kayis S.A et al., 2017 |
| *ACTB* | CGACATCCGTAAGGACCTGT<br>CAGGGCTGTGATCTCCTTCT | 99 | Klein C. et al., 2011 |
| *HPRT* | AATTATGGACAGGACTGAACGG<br>ATAATCCAGCAGGTCAGCAAAG | 121 | Cappelli K et al., 2008 |
